# Advancements in drug discovery: integrating CADD tools and drug repurposing for PD-1/PD-L1 axis inhibition†

**DOI:** 10.1039/d4ra08245a

**Published:** 2025-01-23

**Authors:** Patrícia S. Sobral, Tiago Carvalho, Shiva Izadi, Alexandra Castilho, Zélia Silva, Paula A. Videira, Florbela Pereira

**Affiliations:** a LAQV and REQUIMTE, Departamento de Química, Faculdade de Ciências e Tecnologia, Universidade Nova de Lisboa Caparica Portugal florbela.pereira@fct.unl.pt; b UCIBIO, Departamento Ciências da Vida, Faculdade de Ciências e Tecnologia, Universidade Nova de Lisboa Caparica Portugal zm.silva@fct.unl.pt p.videira@fct.unl.pt; c Associate Laboratory i4HB – Institute for Health and Bioeconomy, NOVA School of Science and Technology, Universidade NOVA de Lisboa 2829-516 Caparica Portugal; d CDG & Allies – Professionals and Patient Associations International Network (CDG & Allies – PPAIN), Department of Life Sciences, NOVA School of Science and Technology, Universidade NOVA de Lisboa 2829-516 Caparica Portugal; e University of Natural Resources and Life Sciences, Department of Applied Genetics and Cell Biology Vienna Austria

## Abstract

Despite significant strides in improving cancer survival rates, the global cancer burden remains substantial, with an anticipated rise in new cases. Immune checkpoints, key regulators of immune responses, play a crucial role in cancer evasion mechanisms. The discovery of immune checkpoint inhibitors (ICIs) targeting PD-1/PD-L1 has revolutionized cancer treatment, with monoclonal antibodies (mAbs) becoming widely prescribed. However, challenges with current mAb ICIs, such as limited oral bioavailability, adverse effects, and high costs, underscore the need to explore alternative small-molecule inhibitors. In this work, we aimed to identify new potential ICI among all FDA-approved drugs. We employed QSAR models to predict PD-1/PD-L1 inhibition, utilizing a diverse dataset of 29 197 molecules sourced from ChEMBL, PubChem, and recent literature. Machine learning techniques, including Random Forest, Support Vector Machine, and Convolutional Neural Network, were employed for benchmarking to assess model performance. Additionally, we undertook a drug repurposing strategy, leveraging the best *in silico* model for a virtual screening campaign involving 1576 off-patent approved drugs. Only two virtual screening hits were proposed based on the criteria established for this approach, including: (1) QSAR probability of being active against PD-L1; (2) QSAR applicability domain; (3) prediction of the affinity between the PD-L1 and ligands through molecular docking. One of the proposed hits was sonidegib, an anticancer drug, featuring a biphenyl system. Sonidegib was subsequently validated for *in vitro* PD-1/PD-L1 binding modulation using ELISA and flow cytometry. This integrated approach, which combines computer-aided drug design (CADD) tools, QSAR modelling, drug repurposing, and molecular docking, offers a pioneering strategy to expedite drug discovery for PD-1/PD-L1 axis inhibition. The findings underscore the potential to identify a wider range small molecules to contribute to the ongoing efforts to advancing cancer immunotherapy.

## Abstract 2

A pioneering strategy to expedite drug discovery for PD-1/PD-L1 axis inhibition is offered by an integrated approach combining computer-aided drug design (CADD) tools, QSAR modelling, drug repurposing, and molecular docking.

## Graphical abstract

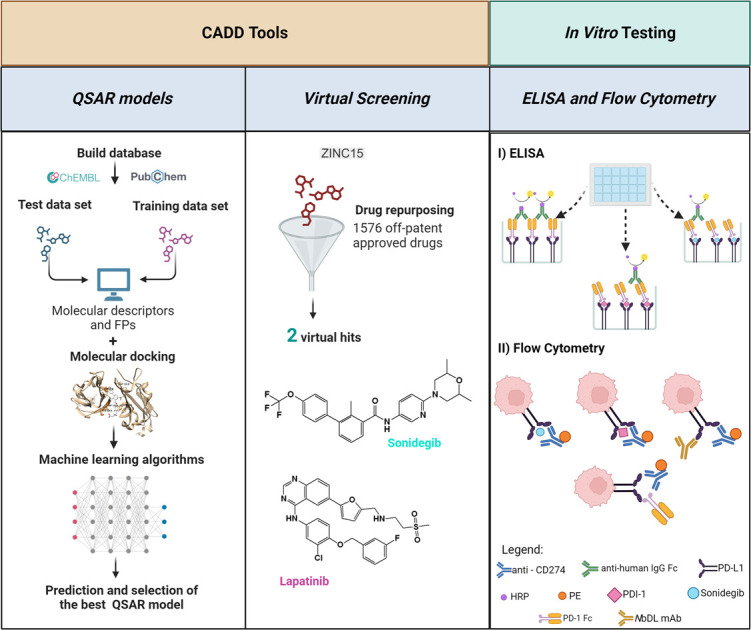

## 1. Introduction

Notwithstanding significant improvement in relative survival rates, cancer continues to be the leading or second leading cause of death globally.^1^ Additionally, an estimated 29.9 million new cancer cases per year are predicted to occur in 2040, marking a 33% increase from the 20 million cases reported in 2022.^2,3^

Cancers are characterized by countless genetic and epigenetic alterations that produce a variety of tumor antigens. The immune system can exploit these alterations to recognize tumor cells and activate effector T cells to fight the tumor. In a healthy individual, immune checkpoints are key to controlling the action of T cells, and for protecting tissues in response to pathogenic infections or auto-immunity. However, in the presence of tumors, the expression of these proteins can become dysregulated. This dysregulation can make cancer cells undetectable and diminishes their elimination by cytotoxic T cells, allowing them to grow.^4^ One way to overcome this resistance mechanism involves utilizing antibodies, small molecules or receptors that will act as immune checkpoint blockers or modulators. This approach is effective because most immune checkpoints are activated through ligand–receptor interactions.^5^ PD-1 is a transmembrane glycoprotein belonging to the immunoglobulin (Ig) superfamily, consisting of 288 amino acids. It consists of a solitary N-terminal IgV-like domain, an approximately 20 amino acid stalk that separates the IgV domain from the plasma membrane, a transmembrane domain, and a cytoplasmic tail housing tyrosine-based signaling motifs. In contrast, PD-L1 features a transmembrane region and two extracellular domains, IgC and IgV. The short cytoplasmic domain of PD-L1 initiates intracellular signaling pathways.^6^ Activated T cells, B cells, dendritic cells, and natural killer cells express high levels of PD-1, while its ligand, PD-L1, is expressed on various types of tumor cells.^5,6^

The clinical translation of immune checkpoint inhibitors (ICIs), drugs that modulate T cell activation, was unquestionably the greatest accomplishment in cancer treatment in the last decade. This breakthrough began in 2011 with the approval of ipilimumab, the first antibody blocking the immune checkpoint Cytotoxic T-lymphocyte associated protein 4 (CTLA4). Next, pembrolizumab and nivolumab were developed, targeting PD-1, along with durvalumab and atezolizumab, which target PD-L1. So far, eight agents have been approved as PD-1/PD-L1 immune checkpoint inhibitors (Table S1 available in the ESI†).^6–9^

**Fig. 1:**
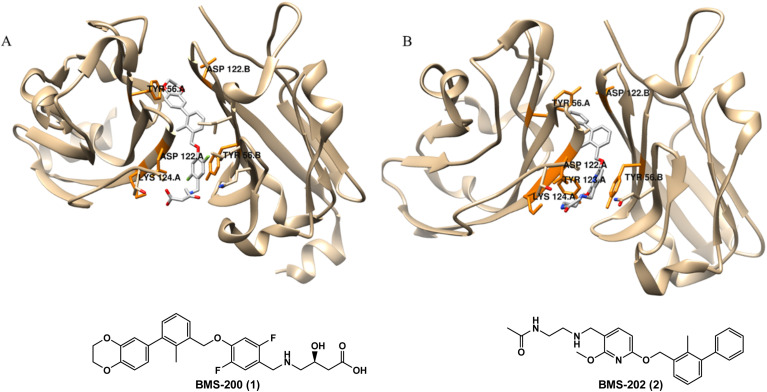
The co-crystal dimer structure of PD-L1 in complex with (A) BMS-200 (1) (Protein Data Bank, PDB ID: 5N2F) and (B) BMS-202 (2) (Protein Data Bank, PDB ID: 5J89) is highlighted, illustrating the critical residues for ligand binding.

While approved ICIs are currently monoclonal antibodies (mAbs), they have drawbacks such as limited oral bioavailability, extended tissue retention, suboptimal membrane permeability and high costs. Consequently, research focus has shifted towards creating small molecule inhibitors to overcome these constraints associated with mAbs.^9^ The interaction between PD-1 and PD-L1 receptors is a typical example of protein–protein interaction (PPI), where the binding sites are shallow and poorly defined, and are generally too large (∼1970 Å^2^ for PD-1/PD-L1) to accommodate a small molecule. This makes designing inhibitors for such interactions particularly difficult.^10,11^ In 2015, the examination of PD-1/PD-L1 crystal structures, combined with molecular network mapping, led to the discovery of potential hotspots. Three key regions on PD-L1 were identified: a hydrophobic cleft containing Met115, Ala121, and Tyr123; a hydrophobic pocket composed of the side chains of Tyr56, Glu58, Arg113, Met115, and Tyr123; and an elongated groove involving the main chain and side chains of Asp122, Tyr123, Lys124, and Arg125. All these regions are considered suitable for small molecule binding to PD-L1.^10,11^ Also in 2015, Bristol-Myers Squibb (BMS) disclosed the first small molecules exhibiting promising inhibitory activity against PD-L1. These molecules comprise a series of compounds featuring a biphenyl group.^12^ Subsequently, Holak's group elucidated the binding mechanism, revealing that the BMS compounds induced the dimerization of the PD-L1 protein. The disclosure of two co-crystal structures, PD-L1 in complex with small molecule inhibitors BMS-200 (1) and BMS-202 (2) (PDB ID: 5N2F and PDB ID: 5J89, respectively), provided insight into structure-based drug design^13,14^ (Fig. 1).

These findings led to follow-up docking studies and consequently to the development of BMS derivatives that retain the biphenyl moiety. Further evidence confirmed that the residues Tyr56, Asp122 and Lys124 are crucial for ligand binding, following in this case a ligand-based approach.^15^ Although no small molecules have yet been approved as PD-1/PD-L1 ICI to date, six small molecules are currently undergoing clinical trials, predominantly in the early phases,^16^ as outlined in Table S2 available in the ESI.†

**Fig. 2:**
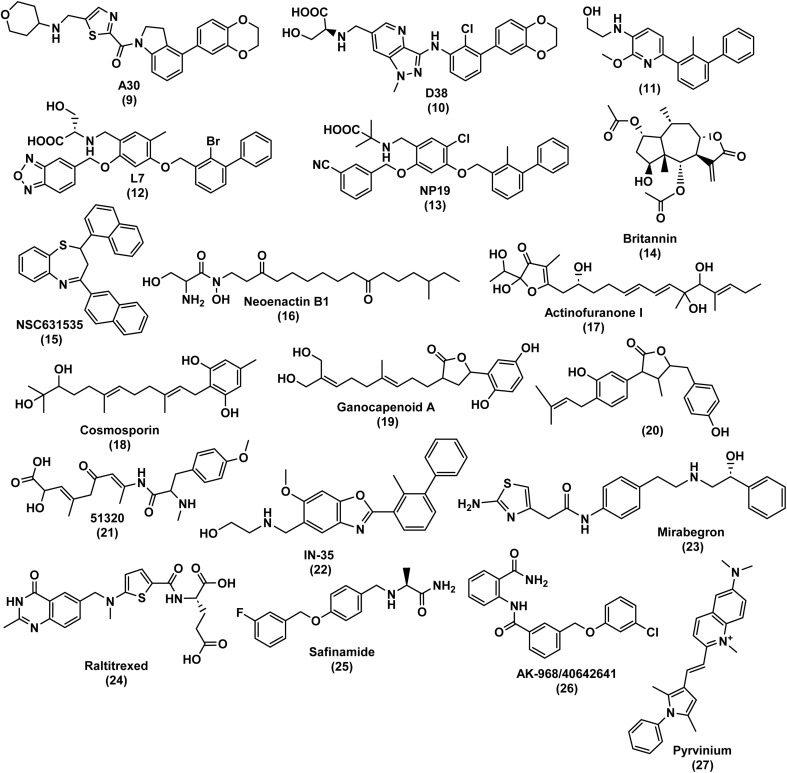
Chemical structures of small molecule inhibitors of the PD-1/PD-L1 immune checkpoint.

A few studies have been reported on Computer-Aided Drug Design (CADD) for inhibitory activity against PD-1/PD-L1,^17^ with some of them simply using a Structure Activity Relationship (SAR) strategy and docking against PD-L1 as a corroboration approach, manly based on the pharmacophoric model of BMS compounds.^18–34^ One of those works was performed by Qin *et al.*,^29–31^ who developed several studies in which numerous compounds were obtained using a ring fusion strategy exploring series of [1,2,4]triazolo[4,3-*a*]pyridines, indolines and 4-arylindolines scaffolds, reaching the compound A30 (9) represented in Fig. 2 with an IC_50_ value of 11.2 nM using the latter scaffolds. Dai *et al.*^32^ obtained compound D38 (10) with an IC_50_ value of 9.6 nM by exploring pyrazolo [4,3-*b*] pyridine derivatives. Wang and his team^34^ obtained a biphenyl pyridine (11) with an IC_50_ value of 3.8 nM, while Liu *et al.*^33^ developed benzo[*c*][1,2,5]oxadiazole derivatives, with the compound L7 (12) presenting an IC_50_ value of 1.8 nM (Fig. 2). Cheng and co-workers^35^ opted to use molecular docking as a starting point, conducting a docking-based virtual screening and drug design based on SARs study of the top hits, resulting in NP19 (13) shown in Fig. 2. NP19 is a resorcinol dibenzyl ether with the same core group as BMS-202, with an IC_50_ value of 12.5 nM. Similarly, Vergoten *et al.*^36^ also employed a molecular docking approach, focusing on pseudoguaianolide sesquiterpene lactones, particularly britannin (14, Fig. 2). They chose britannin due to its known potential as a potent anticancer agent acting *via* modulation of the transcription factor NFkB and the Nrf2-Keap1 signaling pathway, as well as its ability to induce down-regulation of the ICI PD-L1. The computed empirical energy of interaction (Δ*E*) for the BRT-PD-L1 dimer complex was approximately −63.1 kcal mol^−1^, closely resembling the value obtained for the reference PD-L1 ligand BMS-202 (2, Fig. 1) (Δ*E* = −73.4 kcal mol^−1^) under the same conditions.

Though most studies use a more straightforward approach for identification of PD-1/PD-L1 binders, some studies reported the practice of more complex strategies. One such study is by DiFrancesco *et al.*,^37^ which, similar to Cheng's work,^35^ starts with a docking-based virtual screening. The key difference lies in the choice of compound library: Cheng's work utilized Targetmol's natural compound library containing 1867 compounds, whereas DiFrancesco's work involved screening of approximately 3.7 million lead-like molecules from the ZINC repository,^38^ against both human PD-1 and PD-L1. Due to the challenging small molecule tractability of the PD-L1 binding, they opted to continue only with PD-1 and performed another docking-screening, this time using the National Cancer Institute (NCI) dataset. These screenings followed specific criteria: a molecular weight between 250 and 350 g mol^−1^, an *X*log*P* value of ≤3.5 and a maximum of 7 rotatable bonds. From the results, 40 top hits were selected based on factors such as the commercial availability, cost, and binding to key residues of PD-1 and PD-L1. Subsequently, a Molecular Dynamics (MD) simulation with the Desmond Molecular Dynamics package was performed, revealing NSC631535 (15), shown in Fig. 2, as the most promising compound with a IC_50_ value of 15 μM. Similarly, Kumar and his team^39^ also began their study with a docking-based high-throughput virtual screening, utilizing the Natural Product Atlas database against PD-L1. The ligands were also filtered using ADME and drug-likeness criteria, this time using QikProp tool. Following MD simulation, five natural compounds emerged as top hits: neoenactin B1 (16), actinofuranone I (17), cosmosporin (18), ganocapenoid A (19) and 3-[3-hydroxy-4-(3-methylbut-2-enyl)-2-methylidene-cyclohexanone (20) (Fig. 2).

Another commonly employed strategy is the structure-based pharmacophore-based virtual screening (PBVS) approach.^40–44^ Urban *et al.*'s^40^ developed a structure-based pharmacophore model using Pharmit server software, utilizing crystal structures of the PD-L1 dimer in complex with BMS-8 (PDB ID 5J89), BMS-202 (2) (PDB ID 5J8O), BMS-1001 (PDB ID 5NIU) and BMS-1166 (PDB ID 6R3K). Over 90 million compounds from the PubChem database were screened against this model. The matching compounds were then subjected to docking to the PD-L1 dimer using AutoDock Vina software, followed by further screening using QikProp program and SwissADME web tool to the compounds of the complexes with lower energy (kcal mol^−1^). Subsequently, MD simulations were conducted, revealing nine compounds exhibited stable complexes with PD-L1, although their identities were not disclosed. Luo and his team^41^ used a very similar strategy, using Discovery Studio 4.5 to construct the pharmacophore model. Instead of using PubChem, they screened marine small molecules databases such as Comprehensive Marine Natural Products Database (CMNPD) and the Seaweed Metabolite Database (SWMD). Likewise, ADME, toxicity and docking studies were performed using the SwissADME, ProTox-II and CDOCKER programs, respectively. Compound 51320 (21), represented in Fig. 2, was selected for MD analysis, with the results showing a stable binding to PD-L1 and the potential to become an ICI. Surmiak *et al.*^45^ reported a comparison of representative molecules from different classes, such as mAbs, macrocyclic peptides, and small molecules, in terms of their PD-1/PD-L1 dissociation capacity measured by Homogeneous Time-Resolved Fluorescence (HTRF) and their *in vitro* bioactivity assessed through the immune checkpoint blockade co-culture assay. The authors concluded that, unlike mAbs and macrocyclic peptides, most of the known PD-L1 targeting small molecules do not simply block the PD-L1 surface in a 1 : 1 molar ratio. Instead, these small molecules induce homodimerization of human PD-L1 *in vitro*.

Chandrasekaran *et al.*,^42^ Fattakhova *et al.*^43^ and Pushkaran *et al.*^44^ also follow a structure-based PBVS approach, but they complement it with a drug repurposing strategy. Drug repurposing,^46^ also referred to as drug repositioning or reprofiling, entails discovering new applications for approved or investigational drugs beyond their original medical indications. This approach presents several benefits over the development of entirely new drugs. Firstly, there is a lower risk of failure since the repurposed drug has already undergone safety assessments in preclinical models and humans, reducing the likelihood of safety-related failures in subsequent efficacy trials. Secondly, the drug development timeline is abbreviated as a significant portion of preclinical testing, safety evaluation, and, at times, formulation development is already completed. Thirdly, the required investment is reduced, contingent on the stage of development of the repurposing candidate. While regulatory and phase III costs may remain comparable, substantial savings are achievable in preclinical and phases I and II trials. These advantages have the potential to yield a less risky and faster return on investment in the development of repurposed drugs, with lower average associated costs. These costs are estimated to be approximately $300 million on average, in contrast to the $2–3 billion typically associated with developing a new chemical entity.^46^ Chandrasekaran *et al.*^42^ based their pharmacophore model on observed interactions between the PD-L1 dimer and INCB086550 (4), a compound undergoing clinical trials. They identified six key properties: two acceptors of hydrogen bonds, one donor of hydrogen bonds, one positively ionizable group and two aromatic rings. This model, created using PHASE module, was employed to screen FDA-approved drugs. The FDA-approved drugs with the highest scores were compared with a clinical trial candidate, IN-35 (22). Further screening, docking and MD simulations were performed, revealing mirabegron (23) (Fig. 2), a drug approved for overactive bladder, as their top hit. Pushkaran *et al.*^44^ used a similar strategy, using in this case the PD-L1/BMS-202 (2) complex and the “Structure-based pharmacophore” module of the Ligand Scout 4.1 program.^47^ This model was then used for screening all the FDA-approved drugs in the DrugBank database^48^ and small molecules in the Specs database. After docking-screening, *in vitro* studies were performed, revealing that raltitrexed (24), safinamide (25) and specs compound (AK-968/40642641) (26), shown in Fig. 2, effectively increased the proliferation of immune cells and IFN-γ production. Fattakhova and co-workers^43^ opted to start with a docking-screening of ZINC15 database that includes ∼10 000 approved and investigational drugs. The AutoDock Vina docking algorithm was employed for the structure-based docking of drug molecules to multiple PD-L1 dimer interfaces (PDB IDs: 5N2F, 5NIU, 6R3K, 5J89, 5J8O, 5N2D, 6NM8). The selection process involved picking the top 1000 molecules with the most favorable docking scores. Subsequently, the ligand-based virtual screening of ZINC15 utilized ROCS 3.4.1.0, a database ranking drugs based on 3D structure similarity. Compounds with higher Tanimoto Combo scores, indicating greater similarity to seven crystal ligands, were then combined with the initial 1000 molecules. These leading ROCS hits underwent further docking against the high-resolution PD-L1 crystal structure (PDB: 5N2F). After conducting molecular dynamics (MD) analysis and Homogeneous Time-Resolved Fluorescence (HTRF) binding assays, Pyrvinium (27) (Fig. 2), an FDA-approved anthelmintic drug, demonstrated the highest activity with an IC_50_ value of approximately 29.66 μM. The AutoDock Vina docking algorithm was employed for the structure-based docking of drug molecules onto various PD-L1 dimer interfaces (PDB IDs: 5N2F, 5NIU, 6R3K, 5J89, 5J8O, 5N2D, 6NM8). The selection process involved picking the top 1000 molecules with the most favorable docking scores. Following this, the ligand-based virtual screening of ZINC15 utilized ROCS 3.4.1.0, a database ranking drugs based on 3D structure similarity. Compounds with higher Tanimoto Combo scores, indicating greater similarity to seven crystal ligands, were subsequently merged with the initial 1000 molecules.

Here, we employed a combined approach of CADD tools and drug repurposing, adopting a methodology distinct from previously reported ones and akin to our group's prior work. We constructed classification QSAR models utilizing empirical molecular descriptors and fingerprints to predict the inhibition of the PD-1/PD-L1 axis, employing active or inactive labels. A total of 29 197 molecules from the ChEMBL and PubChem databases, along with recent literature from the Web of Science, were utilized to build these models. We explored three machine learning (ML) techniques—Random Forest, Support Vector Machine, and Convolutional Neural Network—to predict PD-1/PD-L1 inhibition, assessing model performance through internal and external validation. Subsequently, utilizing the best *in silico* model, we conducted a virtual screening campaign using 1576 off-patent approved drugs (FDA, EMA, and other agencies) obtained from the ZINC database. Two virtual screening hits, sonidegib and lapatinib, were proposed based on their potential to act as active PD-1/PD-L1 axis inhibitors in the QSAR model, their affinity (kcal mol^−1^) to PD-L1, binding to key residues assessed through docking studies, and the applicability of the top-performing model. Due to solubility issues, only sonidegib was experimentally evaluated. Finally, we confirmed the *in vitro* activity of sonidegib as a PD-1/PD-L1 modulator using an ELISA method and flow cytometry-based competition assays.

## 2. Results and discussion

### 2.1. QSAR classification modelling

The whole data set comprising 29 197 organic molecules that was randomly partitioned based on the two PD-L1 activity classes into a training set of 28 319 molecules (403 active and 27 916 inactive molecules), a test_1 set of 878 molecules (14 active and 864 inactive molecules), and a test_2 set of 1000 molecules (14 active and 986 inactive). These sets were used for the development (training set) and external validation (test set 1) of the QSAR classification models. The test set 2 was used for an additional internal validation. The training set was further categorized into five structural clusters or scaffold types (A–D, and X). Tables 1 and 2 display the five structural clusters along with their centroids, as well as the count of PD-L1 classes (active and inactive) within each structural cluster, and Murcko scaffold analysis. The clustering and Murcko scaffolding were done using Data Warrior.^49^ The Tanimoto coefficient of similarity was calculated using an RDKit script.^50^

**Table 1:** Structural clusters and counts of PD-L1 classes for the training set

Cluster^a^	#^b^	PD-L1 classes^c^		*C*Log*P*^d^		MW^e^		Rotatable bonds^f^		Polar surface area^g^ (Å^2^)	
		Active	Inactive	Active	Inactive	Active	Inactive	Active	Inactive	Active	Inactive
A	25 605	299 (1%)	25 306 (99%)	3.72	2.96	573.19	503.37	10.29	6.84	115.37	118.02
B	1644	78 (3%)	1566 (97%)	4.21	2.89	465.56	340.63	9.00	4.36	75.92	81.11
C	101	8 (8%)	93 (92%)	2.96	3.74	232.29	305.44	2.5	2.84	29.27	47.20
D	26	16 (62%)	10 (38%)	−8.24	0.95	1712.60	762.74	42.63	13.50	688.10	228.70
X	943	2 (0%)	941 (100%)	4.12	2.22	398.50	255.71	8.5	3.94	60.84	60.86

a Cluster code.

b Number of molecules.

c Within the cluster for the training set.

d Average value of *C*Log*P* (an estimation of Log*P*, the octanol–water partition coefficient), within the category for the training set.

e Average value of MW (molecular weight) within the category for the training set.

f Average value of Rotatable bonds within the category for the training set.

g Average value of polar surface area (Å^2^) within the category for the training set.

**Table 2:** Chemical structure of centroids and their Murcko scaffolds for the five structural clusters in the training set

Cluster^a^	Centroid^b^	Centroid Murcko scaffold^c^	Number of Murcko scaffolds^d^ (%)
A	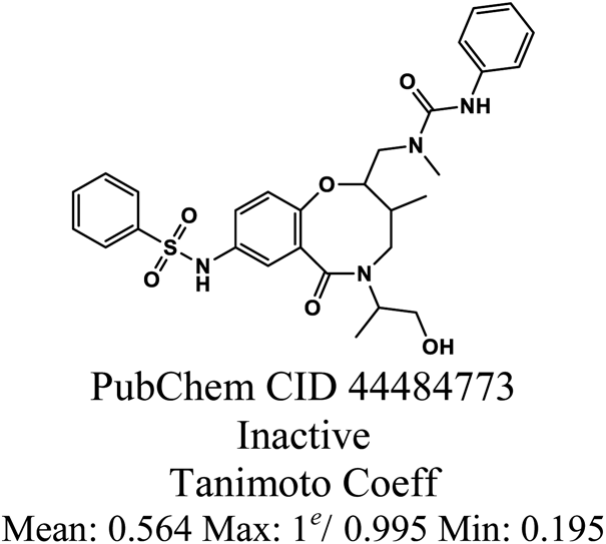	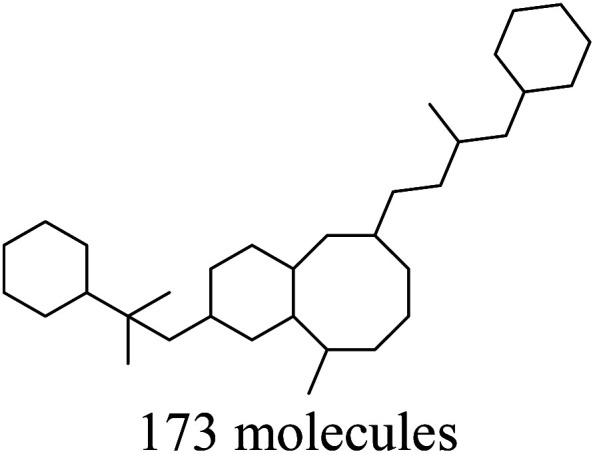	4180 (16%)
B	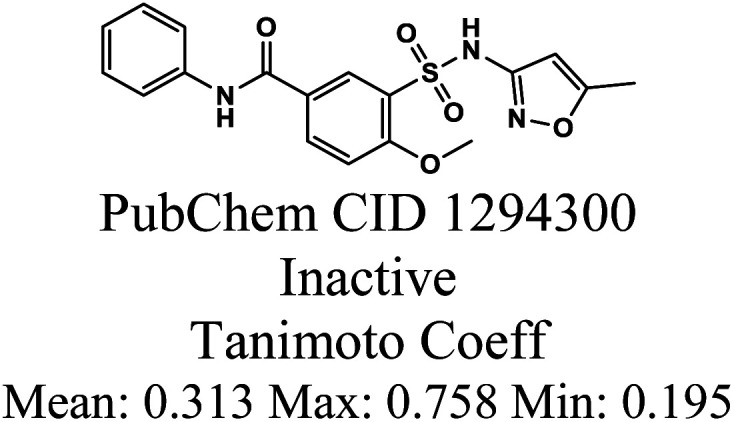	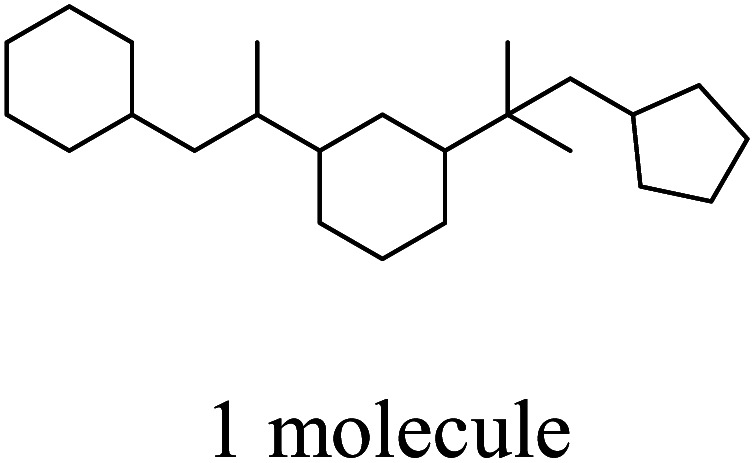	608 (37%)
C	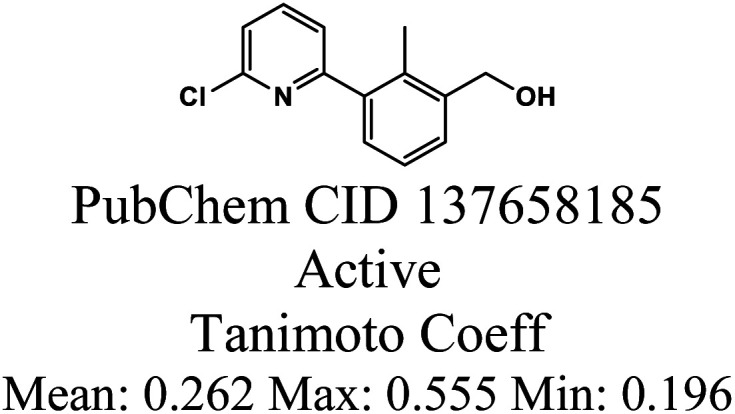	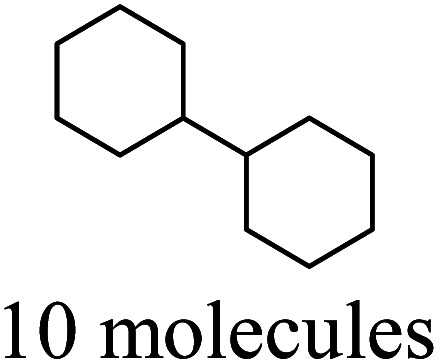	58 (57%)
D	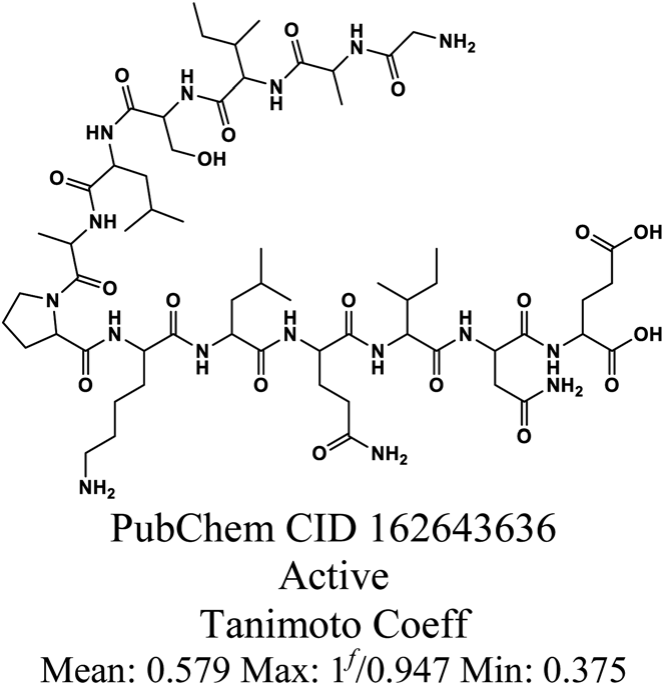	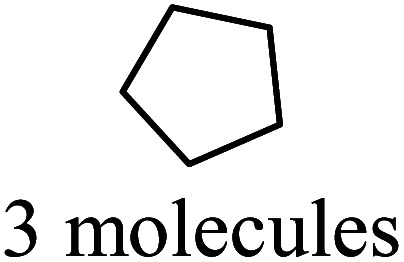	18 (69%)
X	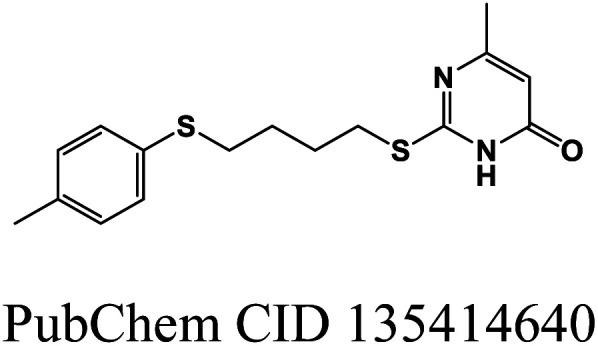	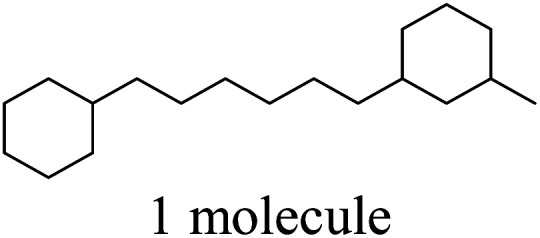	281 (30%)

a Cluster code.

b Chemical structure of the cluster centroid.

c Chemical structure of the centroid Murcko scaffold.

d Percentage within the category for the training set.

e Contains five enantiomers of the centroid.

f Contains one enantiomer of the centroid.

After clustering our training set using Data Warrior, we obtained four clusters (A, B, C, and D). Cluster A contained the majority of the molecules, while cluster D comprised only 26 molecules. Upon analysing the corresponding centroids and the Tanimoto coefficient between them and the remaining molecules in each cluster, we found that the minimum Tanimoto coefficient for clusters A to C ranged from 0.03 (C) to 0.06 (A and B), whereas for cluster D, it was 0.375. To balance the clusters and enhance their internal similarity, we opted to exclude molecules from each cluster with a Tanimoto coefficient below 0.195. These molecules are denoted as category X in Tables 1 and 2. It's clear that the group of excluded molecules mainly consists of inactive molecules, with only two active molecules originally belonging to cluster B. Cluster A continued to be the most representative cluster, with 25 605 molecules as opposed to the previous 26 054.

Upon analysing Table 1, it is possible to observe that the percentage of active class is quite consistent for clusters A, B and C, ranging between 1% to 8%, but significantly higher for the cluster D (62%). Cluster D pertains to peptides, a class of compounds well-known for their significant role as ICIs due to their higher molecular weight (MW) (1712.60 for the active class). Consequently, peptides are theoretically more capable of acting as blockers in the typically larger active sites of proteins.^10^ Docking studies or biological assays solemnly based on interaction might explain the high percentage of the active class, as good druglikeness might not be expected due to the violation of one of Lipinski's rule-of-five (R-o-5),^51^ which typically limits MW to below 500 Da. However, the MW rule is followed by the other three categories in both classes, except for the active class of cluster A, which has a mean value of 573.19 Da. Nonetheless, Veber's rule^52^ suggests that a more effective discrimination between orally active and inactive compounds within a substantial dataset can be achieved by considering the polar surface area and the number of rotatable bonds. Specifically, compounds that meet the criteria of 10 or fewer rotatable bonds and a polar surface area not exceeding 140 Å^2^ are expected to exhibit favourable oral bioavailability. Additionally, Ghose *et al.*^53^ also extended the R-o-5 by proposing a more limited range of Log*P* values within the range of −0.4 to +5.6. All these criteria are followed by the active class of cluster A, and by the other clusters and classes, with the exception of cluster D, which further validates our analysis. While the focus of this work is small molecules, we opted to maintain this category in our dataset to compare their descriptors and their performance.

The structural variability within each of the five categories (A–D, and X) was assessed by examining the number of Murcko scaffolds within each structural category, as outlined in Table 2. Across the five categories, the range of Murcko scaffolds varies from 18 to 4180, indicating a substantial degree of structural diversity. This diversity is represented by a percentage range of 16–69%, where 100% signifies complete structural variability. It is interesting to note that the mean Tanimoto coefficient,^54^ based on fingerprint-similarity (FP-similarity), is higher between the centroid and rest of the cluster for both cluster A and D (∼0.57). However, the percentage of scaffolds is much lower for cluster A compared to cluster D. While cluster D has a much lower representation that should be considered, it seems that Murcko scaffolds can be a less useful tool to apply to peptides.

RDKit was used to calculate fingerprints (FPs) and molecular descriptors, encompassing three different types of FPs with different sizes (166 MACCS; 1024 Morgan, circular fingerprints and 2048 RDKit) and a total of 242 1D & 2D molecular descriptors, including electronic, topological, and constitutional descriptors. The RF ML technique was used for building the QSAR classification models to predict PD-1/PD-L1 inhibition, and the models' performance was successfully evaluated through internal validation (OOB estimation for the training set), as depicted in Table 3. Among the four sets of FPs and descriptors used to build the QSAR classification model, the Morgan FPs exhibited the best performance.

**Table 3:** Evaluation of the predictive performance of FPs and 1D & 2D molecular descriptors for modelling the PD-L1 activity using the RF algorithm for the training set in OOB estimation. The best models are highlighted in bold

Descriptors	#	SE^a^	SP^b^	*Q* c	MCC^d^
1D & 2D	425	0.896	0.999	0.997	0.895
RDKit FPs	2048	0.950	1.000	0.999	0.961
Morgan FPs	1024	**0.983**	**1.000**	**0.999**	**0.976**
MACCS FPs	166	0.935	1.000	0.999	0.950

a Sensitivity, the ratio of true positive to the sum of true positive and false positive.

b Specificity, the ratio of true negative to the sum of true negative and false negative.

c Overall predictive accuracy, the ratio of the sum of true positive and true negative to the sum of true positive, true negative, false positive and false negative.

d Matthews correlation coefficient.

The 3D descriptors were calculated using GUIDEMOL,^55^ an innovative program created in the scope of our research group. In addition to calculating 3D molecular descriptors already implemented in RDKit, GUIDEMOL also generates grid representations of 3D molecular structures using the electrostatic potential or voxels. The results are presented on Tables 4 and 5.

**Table 4:** Exploration of 3D RDKit descriptors for building PD-L1 classification models using the RF algorithm for the training set in an OOB estimation

Descriptors	#	SE^a^	SP^b^	*Q* c	MCC^d^
AUTOCORR3D	80	0.777	0.998	0.995	0.814
MORSE	224	0.700	0.999	0.995	0.812
RDF	210	0.732	0.997	0.994	0.764
WHIM	114	0.650	0.996	0.991	0.660

a Sensitivity, the ratio of true positive to the sum of true positive and false positive.

b Specificity, the ratio of true negative to the sum of true negative and false negative.

c Overall predictive accuracy, the ratio of the sum of true positive and true negative to the sum of true positive, true negative, false positive and false negative.

d Matthews correlation coefficient.

**Table 5:** Exploration of 3D grid descriptors for building PD-L1 classification models using the RF algorithm for the training set in an OOB estimation

Descriptors	#	SE^a^	SP^b^	*Q* c	MCC^d^
Grid of voxel – atomic number	1331	0.402	0.998	0.990	0.553
Grid of voxel – Log*P*		**0.395**	**0.999**	**0.990**	**0.577**
Grid of voxel – MMFF		0.467	0.996	0.988	0.532
Grid of voxel – MR		**0.397**	**0.999**	**0.990**	**0.561**
Grid of voxel – gasteiger		0.434	0.994	0.986	0.462

a Sensitivity, the ratio of true positive to the sum of true positive and false positive.

b Specificity, the ratio of true negative to the sum of true negative and false negative.

c Overall predictive accuracy, the ratio of the sum of true positive and true negative to the sum of true positive, true negative, false positive and false negative.

d Matthews correlation coefficient.

The best set of fingerprints (FPs), Morgan, along with all RDKit 3D descriptors, and the best 3D grid descriptors were selected for additional investigation (see Table 6). The performances of the two models were compared, with the best results belonged to the model of 1024 Morgan FPs (see Table 3), all 3D RDKit descriptors (see Table 4) and Molar Refractivity grid voxel (see Table 5) comprising a total 3008 descriptors. Subsequently, this model was further optimized through descriptor selection, based on the importance assigned by the RF model using the 25, 50, 100 or 150 most important descriptors. The selection of the 50 most important descriptors from the Morgan FPs, 3D RDKit and Molar Refractivity grid voxel descriptors set, used to build the model with the RF, enabled the training of much smaller RF models with even better prediction accuracies (*Q* = 0.999 and MCC = 0.972) than the models trained with the entire set of descriptors (3008 descriptors) for the training set. A comparison of three machine learning (ML) techniques using RF, SVM and dMPL for building the PD-L1 models with the 50 most important descriptors selected by the RF descriptor importance is shown in Table 7. Considering the better performance of the RF technique compared to SVM and dMPL, it was selected as our QSAR model and therefore applied to the subsequent step, the virtual screening.

**Table 6:** Exploration of three model containing 3D grid descriptors for building PD-L1 classification models using the RF algorithm for the training set in an OOB estimation. The best model is highlighted in bold

Descriptors	#	SE^a^	SP^b^	*Q* c	MCC^d^
Morgan FP + RDKit 3D + grid of voxel – Log*P*	3008	0.935	0.999	0.999	0.948
Morgan FP + RDKit 3D + grid of voxel – MR		**0.938**	**1.000**	**0.999**	**0.954**

a Sensitivity, the ratio of true positive to the sum of true positive and false positive.

b Specificity, the ratio of true negative to the sum of true negative and false negative.

c Overall predictive accuracy, the ratio of the sum of true positive and true negative to the sum of true positive, true negative, false positive and false negative.

d Matthews correlation coefficient.

**Table 7:** Exploration of different ML algorithms using the 50 most important descriptors (Morgan FPs, 3D RDKit and Molar Refractivity grid voxel descriptors). The ML technique with the best performance is highlighted in bold

Descriptors	#	SE^a^	SP^b^	*Q* c	MCC^d^
RF	**Tr^e^**	**0.975**	**1.000**	**0.999**	**0.972**
	**Te^f^**	**1.000**	**0.999**	**0.999**	**0.966**
dMPL	Tr^e^	1.000	0.999	0.999	0.954
	Te^f^	1.000	0.998	0.998	0.934
SVM	Tr^e^	0.945	0.998	0.998	0.918
	Te^f^	1.000	1.000	1.000	1.000

a Sensitivity, the ratio of true positive to the sum of true positive and false positive.

b Specificity, the ratio of true negative to the sum of true negative and false negative.

c Overall predictive accuracy, the ratio of the sum of true positive and true negative to the sum of true positive, true negative, false positive and false negative.

d Matthews correlation coefficient.

e Training set.

f Test set.

### 2.2. Analysis of fingerprints and descriptors

A comparison of the top twenty fingerprints (*i.e.* MACCS) and molecular descriptors (*i.e.* 1D, 2D and 3D) selected by descriptor importance of RF used to build the QSAR classification models, is provided in Table 7 and these descriptors were analysed and presented in descending order of importance in Table 8. The twenty fingerprints (FPs), 1D, 2D and 3D molecular descriptors are listed in decreasing order of importance according to the ‘mean decrease accuracy’ parameter. The respective variations between these are given as 9.11–5.18, 4.95–3.09 and 5.58–3.33. Among these, there are more FPs and molecular descriptors that are more relevant in discriminating the active class than the inactive class in the set of the twenty most important fingerprints and molecular descriptors for modelling PD-1/PD-L1 inhibition. More precisely, there are eleven MACCS FPs, three 1D & 2D descriptors, and four 3D descriptors that are more relevant in discriminating the active class, which are highlighted in green in Table 8. However, the majority of FPs and descriptors (5 MACCs FPs, 14 1D & 2D, and 15 3D) are equally important in discriminating both active and inactive classes, as highlighted in yellow in Table 8.

**Table 8:** The twenty most important MACCS FPs, 1D & 2D and 3D descriptors selected in RF classification models^a^

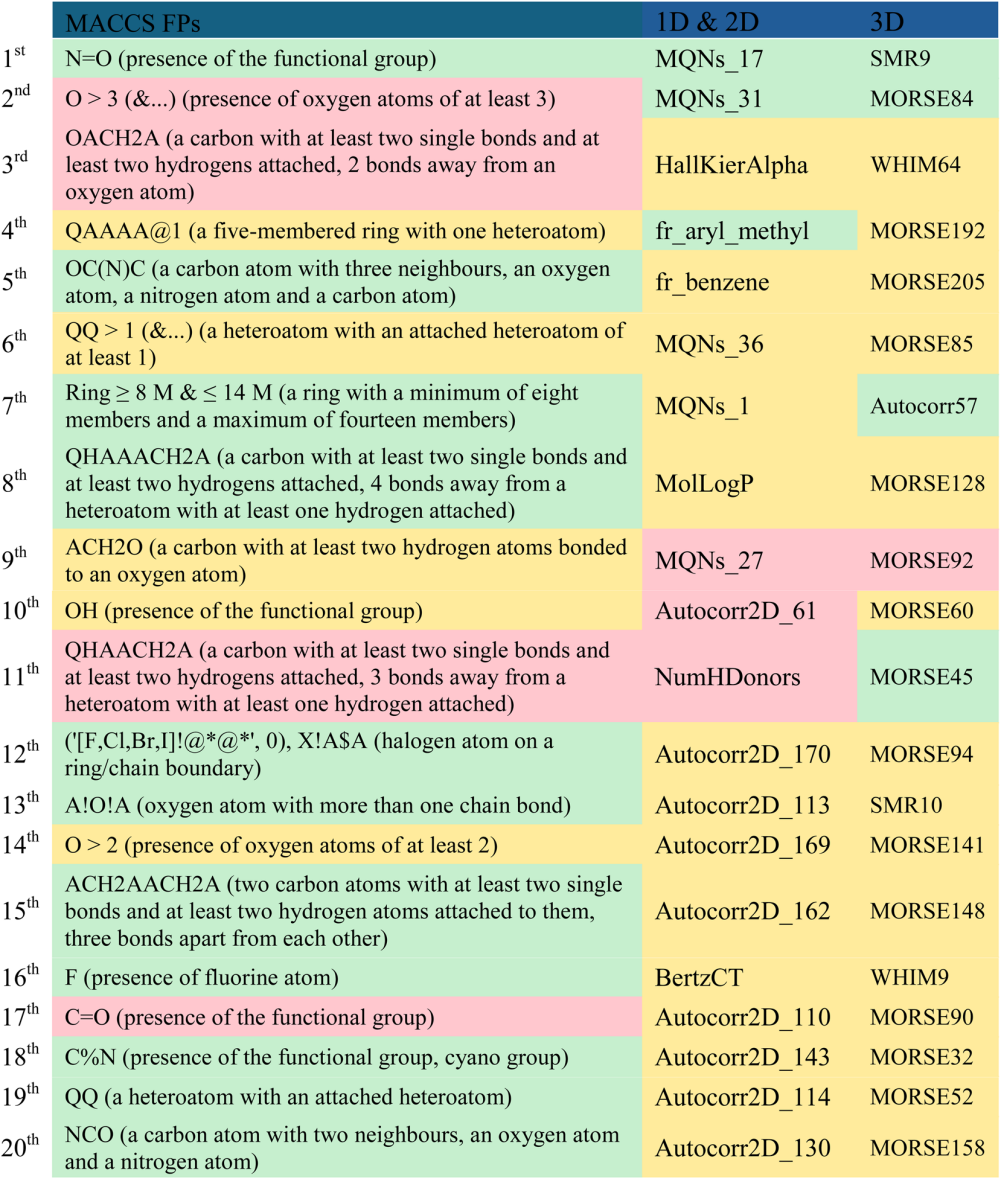

a A – any valid periodic table element symbol; Q – hetero atoms; any non-C or non-H atom; X – halogens; F, Cl, Br, I; % – an aromatic query bond; $ – ring bond; ! – chain or non-ring bond; @ – a ring linkage and the number following it specifies the atoms position in the line, *e.g.* @1 means linked back to the first atom in the list. The FPs and the most relevant molecular descriptors in the discrimination of the active, inactive and both classes were represented in green, red and yellow, respectively.

Considering the MACCS FPs, functionalities like the amide group, lactam ring or the 1,3-oxazole ring can be represented by the 5th and 20th most important MACCS FPs, which are closely associated with the active class. Interestingly, these moieties are present in numerous well-known inhibitors of PD-L1, as highlighted in Fig. 1 (2), Table S2 (3, 4, and 7) available in the ESI, and Fig. 2 (9, 16, 21–26).† Similarly, halogen-based substituents of hydrocarbon rings or derivatives of heterocycles, as well as fluorine substituents, encoded by the 12th and 16th most significant MACCS FPs, respectively, are highly relevant to the active class. In contrast, the hydroxyl substituent encoded by the 10th most important MACCS FPs appears to be relevant for both the active and inactive class. There seems to be a relationship with the number of groups containing oxygen atoms and the activity, as represented in the 2nd most relevant MACCS FP, where oxygen-containing groups greater than 3 appear to be related to the discrimination of the inactive class, as highlighted in red in Table 8.

In the collection of the twenty most significant 1D & 2D descriptors, there are five MQNs (Molecular Quantum Numbers) descriptors,^56^ which encode atom and bond counts, polarity, and topology. The two most significant 1D & 2D descriptors, MQNs_17 and MQNs_31, encode cyclic moieties specifically with double bonds and trivalent nodes, respectively, and are more relevant in discriminating the active class. Conversely, the 9th most important 1D & 2D descriptor, MQNs_27, encodes an acyclic moiety with divalent nodes and is more relevant in discriminating the inactive class. The aryl methyl and phenyl scaffolds, represented by the 4th and 5th most relevant 1D & 2D descriptors, respectively, seem to suggest a distinct activity pattern, with the presence of the methyl group favouring activity. The count of hydrogen donors (*e.g.*, –OH, –SH, –NHR, –HF), represented by the 11th 1D & 2D descriptor, enables the preferential discrimination of the inactive class.

There is a significant majority of MORSE descriptors (Molecule Representation of Structure based on Electron diffraction),^57^*i.e.*, 75%, among the set of the 20 most relevant 3D descriptors in modelling the activity against the PD-1/PD-L1 axis. Specifically, one is an unweighted MORSE descriptor (MORSE32), and the rest are weighted: four, five, three, one, and one MORSE descriptors weighted by relative atomic mass (MORSE45, 52, 57, 60), relative van der Waals volume (MORSE84, 85, 90, 92, 94), relative atomic polarizability (MORSE141, 148, 158), relative atomic ion polarity (MORSE192), and relative I state (MORSE205), respectively. Despite the MORSE descriptor incorporating information about the entire molecular structure, it has been shown that its final value is primarily derived from short-distance atomic pairs (up to 3 Å).^57^ This local effect is even more pronounced with the influence of weighting. It is observed that the most relevant MORSE descriptors for activity against PD-L1 are weighted by atomic mass and van der Waals volume, which significantly decreases the influence of hydrogen and diminishes the roles of nitrogen, oxygen, and fluorine, while increasing the influence of sulfur, chlorine, phosphorus, bromine, and iodine.

### 2.3. Applicability domain of PD-L1 QSAR model

**Fig. 3:**
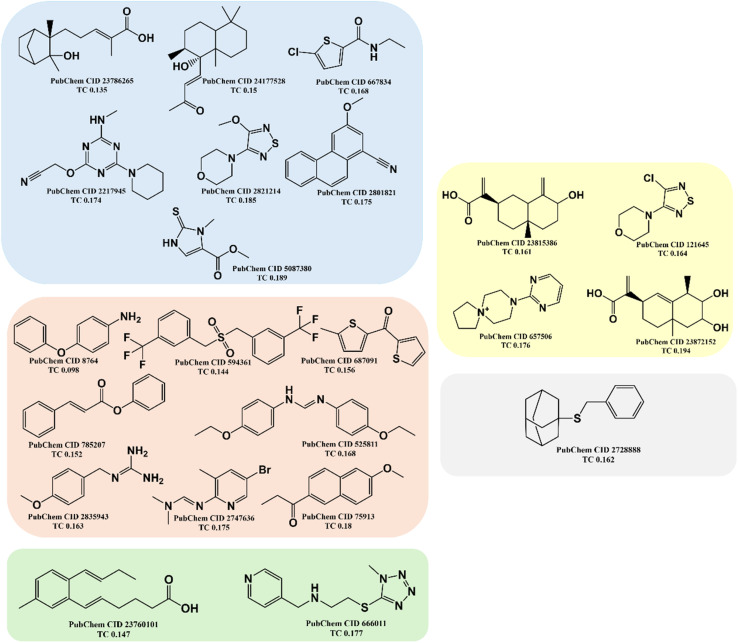
The chemical structures of twenty test set molecules that do not belong to the applicability domain of the PD-L1 QSAR model are shown. Clusters A, B, C, D, and X are highlighted in blue, red, green, yellow, and gray, respectively.

As reported in Section 2.1, the training set was categorized into five structural clusters (A–D, and X), and a centroid was also defined for each of the clusters (see Tables 1 and 2). According to the defined criteria, a given molecule was considered not to belong to the applicability domain of the model if the maximum Tanimoto coefficient obtained from this molecule with the five centroids corresponding to clusters A–D and X was less than 0.195. Applying this threshold, it is found that in the test set there are 22 molecules that do not belong to the applicability domain of the model. All these molecules are predicted as true negatives (TN) and were grouped as follows: seven in cluster A, eight in cluster B, two in cluster C, four in cluster D, and one in cluster X, Fig. 3.

### 2.4. Virtual screening

**Fig. 4:**
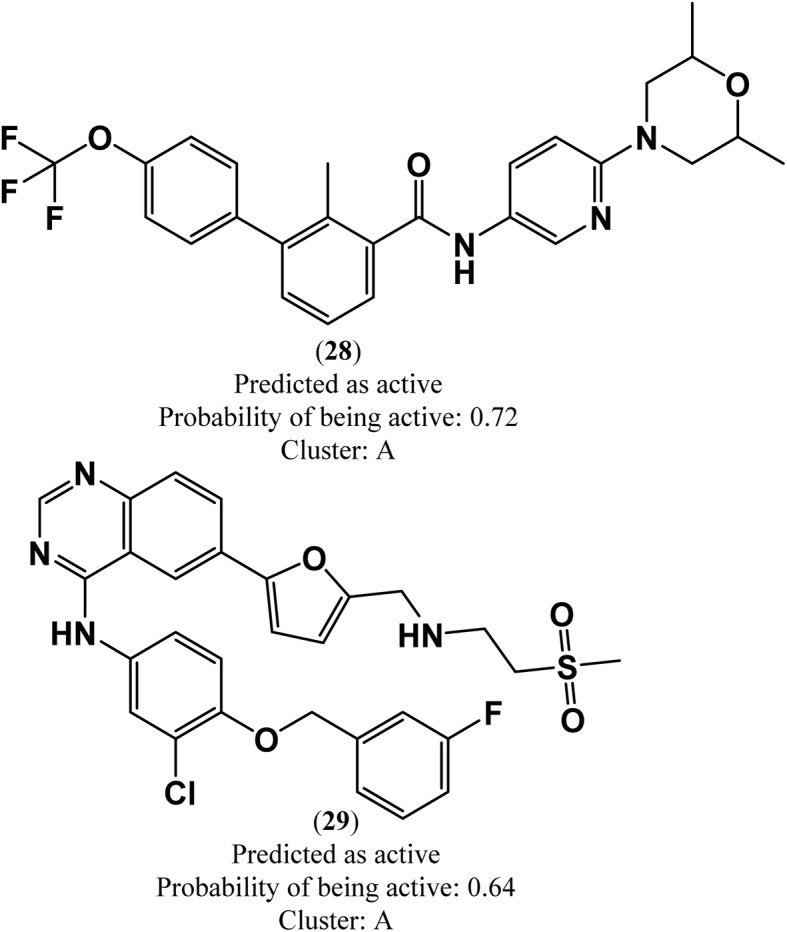
Chemical structures of lead-like PD-L1 inhibitors: sonidegib (28) and lapatinib (29).

In this study, a virtual screening campaign was conducted to identify potential new inhibitors against PD-L1. The best model selected for the virtual screening procedure was the RF classification model, which utilized the 50 most important Morgan FPs, 3D RDKit, and Molar Refractivity grid voxel descriptors. The virtual library consists of 1576 off-patent approved drugs (from FDA, EMA, and other agencies) that are also commercially available compounds. Using the defined threshold for the applicability domain of the PD-L1 model (*i.e.*, belonging to one of the five clusters (A–D, and X) with a maximum Tanimoto coefficient value with the five cluster centroids lower than 0.195), it was possible to prioritize the most probable inhibitors of PD-L1 from the virtual library. Applying this threshold, it was found that 380 molecules in the virtual screening library do not belong to the applicability domain of the model. These molecules were grouped as follows: 109 in cluster A, 34 in cluster B, 25 in cluster C, 167 in cluster D, and 45 in cluster X. The best model identified only two virtual hits from the virtual library of 1196 off-patent approved drugs that belong to the applicability domain of the model and were predicted to be active against PD-L1. These hits, both clustering in category A, were predicted with a probability of being active greater than or equal to 0.64, Fig. 4. In this drug repurposing strategy, two drugs used in cancer treatment were selected as potential candidates: sonidegib (28), a hedgehog signaling pathway inhibitor, and lapatinib (29), a reversible inhibitor of both epidermal growth factor receptor (EGFR) and human epidermal growth factor receptor-2 (HER2) tyrosine kinases, shown in Fig. 4.

### 2.5. Molecular docking

The exploration of PD-1/PD-L1 crystal structures, along with molecular network mapping, led to the identification of potential hotspots on PD-L1 and highlighted three key regions: a hydrophobic cleft composed of Met115, Ala121, and Tyr123; a hydrophobic pocket comprising the side chains of Tyr56, Glu58, Arg113, Met115, and Tyr123; and an extended groove involving the main chain and side chains of Asp122, Tyr123, Lys124, and Arg125. All these regions are considered suitable for small molecule binding to PD-L1.^10,11^

Molecular docking was employed to identify the most favourable binding interactions, and the calculated free binding energies based on the specified search space coordinates are presented in Table 9. This includes the two resulting virtual screening hits—sonidegib (28) and lapatinib (29) as shown in Fig. 4—along with the positive control, BMS-200 (1) as shown in Fig. 1, in accordance with QSAR modelling.

**Table 9:** The calculated free binding energies (Δ*G*_B_, in kcal mol^−1^) and the detailed interactions observed upon docking the two resulting virtual screening hits, sonidegib and lapatinib, as well as the positive control, BMS-200, against PD-L1

#	Name	Δ*G*_B_^a^	Interaction		
			Hydrophobic residues	H-bond residues	π-stacking residues
1	BMS-200	−11.5	Ile54^c^, Tyr56^c^, Met115^c^, Ala121^c^, Ala121^b^, Tyr123^b^	Lys124^b^	Tyr56^c^
28	Sonidegib	−11.0	Ile54^b^, Tyr56^b^, Met115^b^, Met115^c^, Ala121^b^, Ala121^c^, Tyr123^c^	Gln66^b^, Ala121^c^	—
29	Lapatinib	−11.8	Tyr56^b^, Tyr56^c^, Met115^b^, Ala121^c^, Tyr123^b^, Tyr123^c^	Asn63^c^, Gln66^c^	Tyr123^b^

a In kcal mol^−1^.

b Amino acid residues of Chain A.

c Amino acid residues of Chain B.

**Fig. 5:**
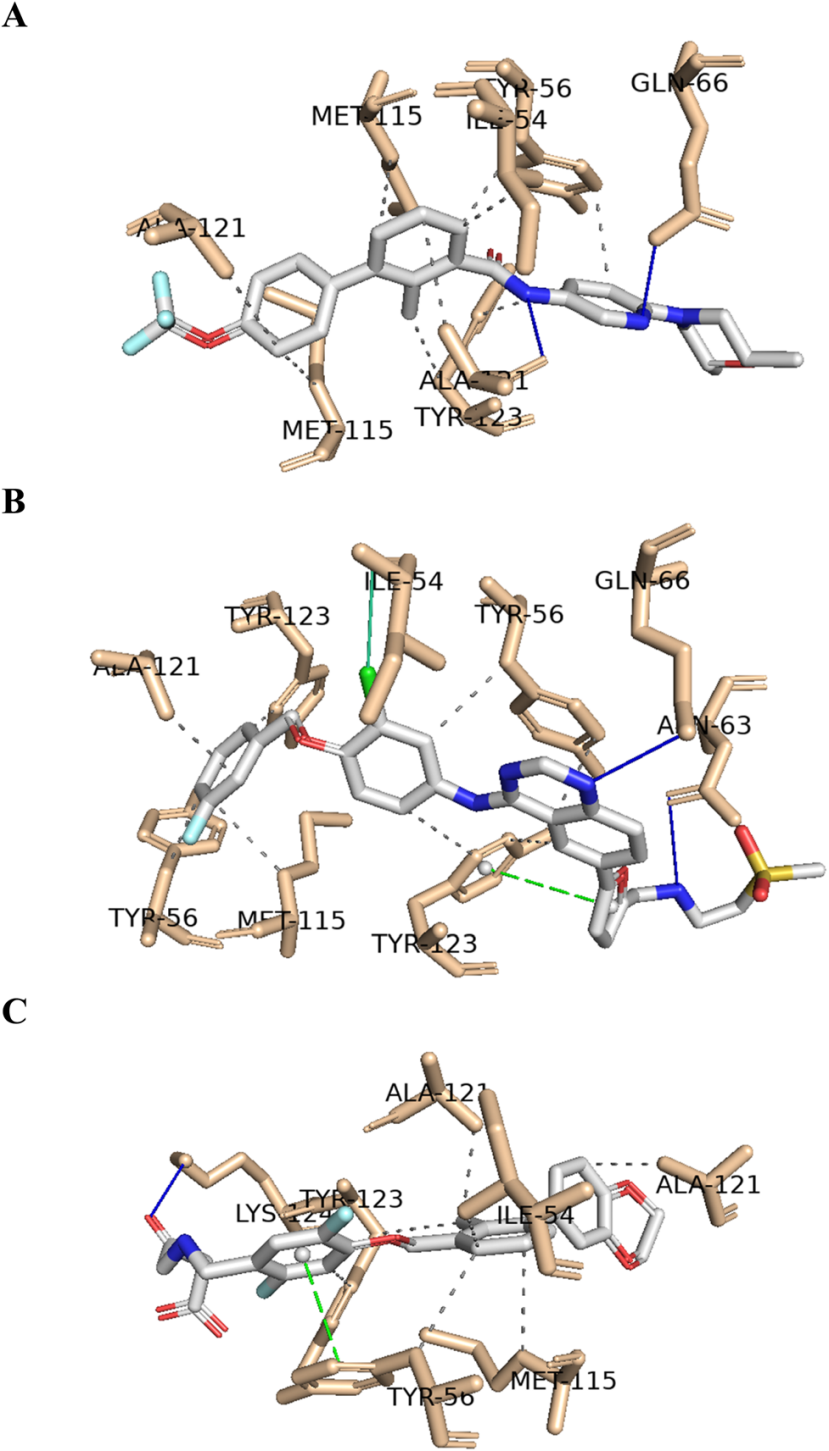
Predicted binding poses of the two hits and positive control in the binding site of PD-L1 (sonidegib – (A); lapatinib – (B); and positive control – (C)). The hydrophobic interactions are shown as black dash lines and the π-stacking interactions in green (parallel) and gray (perpendicular) dash lines. H-bond and halogen-bond interactions are shown as blue and green continuous lines, respectively.

As shown in Table 9, the two resulting virtual screening hits, sonidegib (28) and lapatinib (29), along with the positive control (1), exhibited calculated Δ*G*_B_ values less than or equal to −11 kcal mol^−1^, specifically −11.0, −11.8, and −11.5 kcal mol^−1^, respectively. These excellent binding affinities can be attributed to potential hydrophobic interactions, hydrogen bonds, and π-stacking interactions with key residues in chains A and B of the PD-L1 protein. In Fig. 5, the best-docked poses for the two resulting virtual screening hits, sonidegib and lapatinib, as well as the positive control, BMS-200, are shown.

**Fig. 6:**
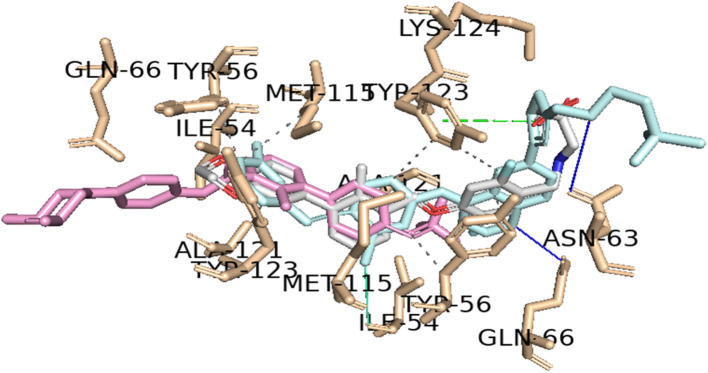
Interaction profiles of the best-docked poses for the sonidegib (pink), lapatinib (blue) and positive control (gray). The hydrophobic interactions are shown as black dash lines and the π-stacking interactions in green (parallel) and gray (perpendicular) dash lines. H-bond and halogen-bond interactions are shown as blue and green continuous lines, respectively.

It is worth noting that both virtual screening hits, sonidegib (28) and lapatinib (29), exhibit a system of four or more rings, similar to the positive control, BMS-200 (1). Sonidegib, like BMS-200, features a biphenyl system. Lapatinib presents a biaryl system and has a binding pose very similar to the positive control, sharing interactions with numerous residues, including Tyr56, Asp122, Tyr123, and Gln66 (see Fig. 6). These residues play important roles in ligand binding to PD-L1, as previously mentioned.

### 2.6. Binding inhibition

#### 2.6.1. Competitive ELISA

**Fig. 7:**
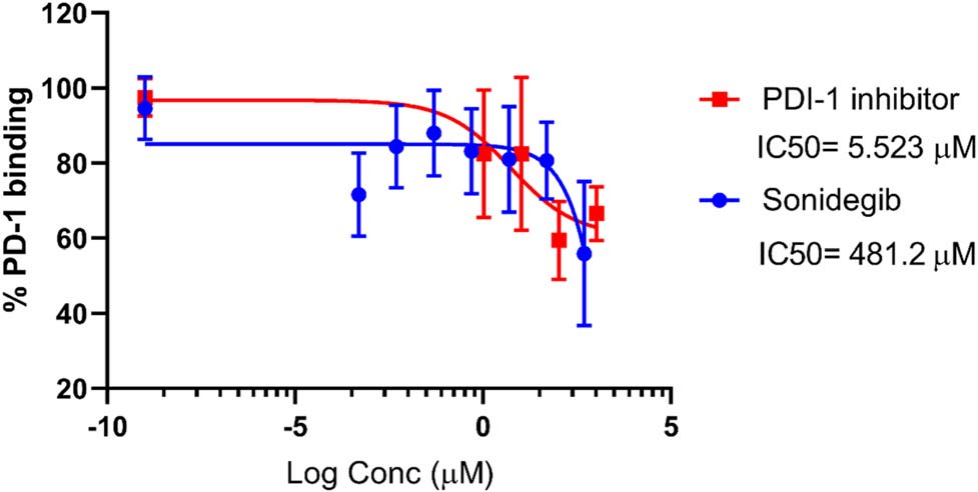
Binding activity of soluble recombinant PD-1Fc to immobilized recombinant PD-L1 in the presence of sonidegib (blue) and of PDI-1 inhibitor (red, positive control) as assessed by competitive ELISA. Graphs represent binding activity (values were normalized to percentage of PD-1/PD-L1 interaction, considering 100% the binding without inhibitors) *vs.* log inhibitor concentration (μM). Values are means ±SD of at least three independent experiments. IC_50_ are indicated.

As described in Material and methods section, purified recombinant human PD-1 and PD-L1 molecules were used to assess binding inhibition by competitive ELISA assay. The ability of small molecules to bind to PD-1 or PD-L1 was tested, and the known PD-L1 inhibitor, PDI-1, was used as positive control. In these assays we coated the plates with 10 μg μL^−1^ of PD-L1 and used 15 μg μL^−1^ of PD-1 (which corresponds to saturating concentration for the tested conditions). The results presented in Fig. 7 show the behaviour of tested molecules in interaction with PD-1/PD-L1 axis. Positive inhibitor control (PDI-1) showed an initial 17.5% of inhibition at 1.0 μM. Sonidegib, in turn, showed an initial 28.4% of inhibition at the minimal concentration of 0.0005 μM. Considering dose–response curves obtained by these results, PDI-1 inhibitor and sonidegib showed a 50% of inhibition at 5.523 μM and 481.2 μM, respectively.

#### 2.6.2. Sonidegib potential inhibition of binding of mAb anti-PD-L1 to cell surface

**Fig. 8:**
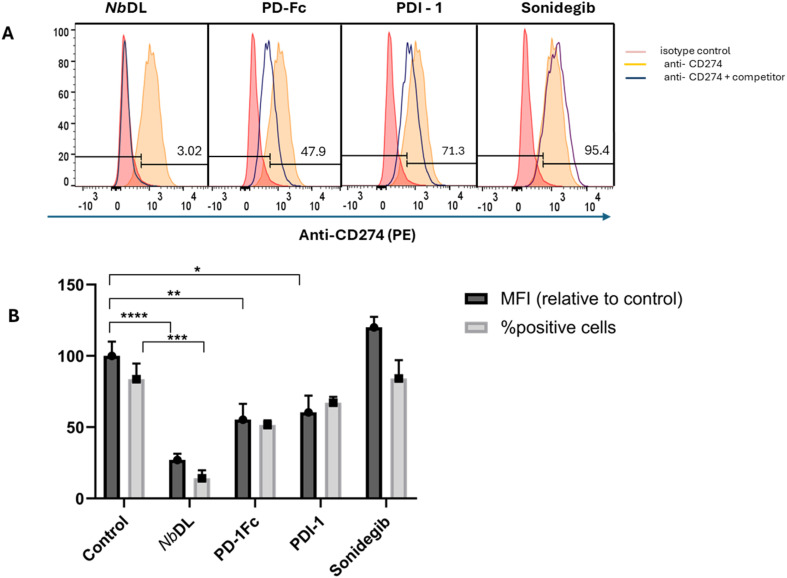
Binding of monoclonal antibody anti-CD274 clone MIH1 to PD-L1 displayed at the surface of the cancer cell line MDA-MB-231. The results show the ability of the represented molecules to interfere with the binding to PD-L1 and were assessed by flow cytometry-based competition assays. (A) Representative histograms showing the binding of mAb anti-CD274 to MDA-MB231 cells, and (B) values of staining of MDA-MB-231with anti-CD274. Results presented as MFI (mean ± SE) normalized to respective control without inhibitor (100%) and as % of positively stained cells (mean ± SE) are from 3 to 5 independent assays. One way ANOVA was applied to assess the statistical significance of differences between multiple treatment groups. ****: *p* < 0.0001, ***: *p* < 0.001, **: *p* < 0.01, *: *p* ≤ 0.05.

To assess if sonidegib has the potential to be used as adjuvant of ICIs mAbs of PD-L1-PD-1 axis, without interfering with ICIs activity, we investigated if sonidegib binding to PD-L1 competes with the sites bound by the ICI anti-PD-L1 mAb (mouse anti-human CD274, clone MIH1). We used the breast cancer cell line MDA-MB-231 due to the high levels of PD-L1 expression (shown in ESI Fig. S1†). To do so, we mixed the fluorescently labelled mAb clone MIH1 with possible competitors. The cells were incubated with the mAb alone or with mAb mixed with sonidegib (500 μM) in the same conditions. In parallel, as positive controls, *Nicotiana benthamiana*-derived durvalumab variant (mAb NbDL) (2 μg mL^−1^), PDI-1 (21 μM) and PD-1Fc (3.4 μg mL^−1^) were used for comparison. The molecule with higher ability to compete with binding of the mAb clone MIH1 was the mAb NbDL (27.1 ± 4.3% relative MFI, 14.1 ± 5.6% of positive cells), followed by PD-1Fc (55.3 ± 11.1% relative MFI, 51.5 ± 3.3% of positive cells) and then PDI-1 (60.4 ± 11.8% relative MFI, 67.2 ± 4.2% of positive cells) (Fig. 8). On the other hand, sonidegib did not significantly interfere with the binding (120 ± 7.4% MFI, 84.2 ± 11.8% of positive cells). These results show that sonidegib is not able to displace the binding of mAb clone MIH1 most probably because it binds to different sites on the PD-L1 molecule. It is not unusual that small molecules occupy different binding sites in PD-L1 when compared to ICIs mAbs. That is the case of BMS-202 and BMS-8, that, although being responsible for the blockade of PD-1/PD-L1 interaction, bind to non-overlapping sites to those of durvalumab VL domain.^58^ Another example is the binding of small molecule PDI-1 compared to that of nivolumab. While, according to modelling of PD-L1 docking, the probable PDI-1 ligation sites in hPD-L1 are Phe 19 and Ser 57 in hPD-1,^59^ nivolumab binds to a N-terminal loop in hPD-1.^60^ In our experimental setting, PDI-1 was able to prevent around 40% the ligation of the mAb clone MIH1, indicating a probable partial overlap to the mAb target sites.

The dimeric PD-1 Fc, although competing for the mAb clone MIH1 binding, only inhibits 45% of the total binding sites. From these observations, we are led to conclude that there is some overlapping in the ligations for PD-1 and the mAb clone MIH1. The ability of NbDL mAb to bind PD-L1 and antagonize the PD-L1-PD-1 binding is well documented.^61^ Moreover, durvalumab has a higher binding affinity compared to that of mAb clone MIH, which may explain its great inhibition.^62^ Importantly, the antagonist blocking effect can be related to having the same binding interfaces and might not depend on having exactly same specific binding sites.^45^

Comparison of the mechanisms of PD-L1–PD-1 blockade *in vitro* have shown that while therapeutic mAbs ICIs bind to target extracellular domains and act as antagonist of the natural ligand, there are other molecules such as the biphenyl-based small molecules that cause the dimerization of the PD-L1 and promote their internalization and degradation.^45,63^ Additionally for another small molecule, the amino acid inspired compound CA-170, a novel mechanism of action was proposed as it does not bind to either PD-L1 or PD-1 directly. The authors suggest that the molecule binds to the already formed PD-L1/PD-1 complex,^64^ creating a “defective ternary complex” that disables this immune checkpoint.

In virtue of their size, small molecules have an intrinsic potential ability to penetrate the cells and target intracellular components which presents one of their great advantages over mAbs as ICIs.^65^ Given the evidence presented here, it is worth to pursue further studies to fully depict sonidegib's molecular interactions, functional activity and mechanisms of action.

## 3. Conclusion

The results suggest that the CADD approach, which combines ligand- and structure-based methodologies and is supported by preliminary experimental evaluation, could be used to predict new PD-1/PD-L1 axis inhibitors from FDA-approved drugs without prior PD-1/PD-L1 activity records. This approach could help identify and propose lead compounds for developing new drugs with potential in cancer immunotherapy. Based on its observed interactions with PD-L1, sonidegib shows potential as a modulator of the PD-1/PD-L1 axis. Although the full extent of its interaction at the cancer cell level has not been thoroughly studied, sonidegib appears to bind to different sites on PD-L1 compared to mAb binding and therefore can be proposed as adjuvant of mAb actions. Further research is required to better understand sonidegib's effects on cancer cells and to pinpoint the specific sites where the molecule exerts its action. Additionally, more in depth mechanistic and functional assays, using cell-based assays and animal model *in vivo* assays will help to ascertain sonidegib's potential ability to be use as immune checkpoint inhibitor.

## 4. Material and methods

### 4.1. Datasets: training and test sets

More than 29 000 small organic molecules were extracted from several curated databases, such as ChEMBL (https://www.ebi.ac.uk/chembl/),^66^ PubChem (https://pubchem.ncbi.nlm.nih.gov/)^67^ and recent literature, through searches based on activity records against the PD1/PDL1 checkpoint receptors. The search was carried out in October 2022, and the following search options were used according to the databases used: ChEMBL (“PD-L1” or “PD-1/PD-L1” ↔ Targets ↔ Associated bioactivities ↔ csv file) and PubChem (“PD-L1” or “PD-1/PD-L1” ↔ Proteins ↔ Chemicals and Bioactivities ↔ csv file). The data set comprises 29 805 organic molecules, namely 29 763 from PubChem database, 40 from ChEMBL database and 2 from literature. After collecting these datasets, duplicates were removed based on the IUPAC international chemical identifier (InChI) codes with consideration for chirality, using the software program OpenBabel (version 2.3.1). For duplicates with different activity values, the respective bioassays were consulted to align the “active label” provided by the assays. For instance, if a tested substance is designated as a chemical probe, active, inactive, inconclusive, or unspecified in an experiment, furthermore, the compounds that were subjected to the aforementioned type of bioassay were also selected. This curation process yielded a total of 29 197 small organic molecules, among which only 417 exhibited activities. The JChem Standardiser tool version 21.9 (ChemAxon Ltd, Budapest, Hungary) was used to standardise molecular structures by normalising tautomeric and mesomeric groups, aromatise and by removing small, disconnected fragments. Three-dimensional models of the molecular structures were generated with JChem CXCALC (JChem 22.11, 2022, ChemAxon Ltd, Budapest, Hungary).

The dataset was divided into two training sets, a training set 1 of 28 319 molecules and a training set 2 of 27 319 molecules, and two test sets, a test set 1 and a test set 2, comprising 878 and 1000 organic molecules, respectively. The latter train and test set 2, were used to validate the Artificial Neural Network and the Support Vector Machine models. The approximate partition of 1:0.03 for training and test sets, respectively, was carried out randomly to ensure that both active and inactive PD-L1 activity classes were adequately represented in both sets, capturing the biological diversity of the dataset.

The built QSAR models were developed and externally validated using the training and test sets, respectively.

The virtual data set consisted of 1576 off-patent approved drugs (FDA, EMA and other agencies), which are also commercially available compounds. The virtual data set consisted of 1576 off-patent approved drugs (FDA, EMA and other agencies), which are also commercially available compounds. These drugs were extracted from the ZINC database (https://zinc.docking.org/) in the SMILES data format using the following search options: Catalogs ↔ Approved Drugs ↔ Extrated ↔ smi file. SMILES strings of the data sets, along with the corresponding experimental and predicted probabilities of being active, are available as ESI, Tables S3–S7.†

The protein images were created using UCSF Chimera 1.16 and the chemical structures using ChemDraw 22.00.

### 4.2. Calculation of descriptors

Empirical molecular fingerprints (FPs) and 1D & 2D molecular descriptors were calculated for the datasets, using RDKit.^50^ Various types of FPs with different sizes were calculated and explored, including 166 MACCS (MACCS keys), 1024 CDK (circular fingerprints) and 2048 RDKit (RDKit fingerprints).^50^ The 1D & 2D molecular descriptors comprised 242 descriptors, containing electronic, topological, and constitutional descriptors.^50^

**Fig. 9:**
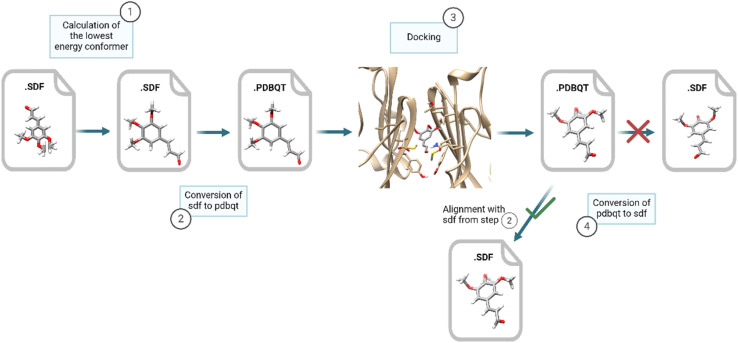
Workflow for 3D optimization: the process of generating SDF files for calculating 3D descriptors required aligning the prior docking SDF files with those generated after docking to retain the coordinates of the latter. The processing steps for a molecule labeled as inactive from the training set are depicted as an example.

As elaborated further ahead, molecular docking against PD-L1 protein was performed on the 29 197 small molecules from the entire dataset. The optimal docking conformation for each molecule, obtained by aligning the original prior-docking SDF files, calculated with JChem CXCALC, with the SDF files obtained as output from docking, was used to calculate the 3D descriptors (Fig. 9). Several well-established 3D molecular descriptors were exploited, such as 3D RDKit descriptors (*e.g.* WHIM, MORSE), alongside the novel 3D grid descriptors. These innovative 3D grid descriptors were calculated using GUIDEMOL, a Python-based computer program built on the RDKit software. GUIDEMOL is designed to process molecular structures and calculate molecular descriptors developed within the framework of the DCMatters project.^55^ Besides calculating 3D molecular descriptors implemented in RDKit, it also generated grid representations of 3D molecular structures using the electrostatic potential or voxels. For instance, it produced grids such as grid of potential – MMFF, grid of voxel – Log*P*.

### 4.3. Optimization of QSAR models: descriptors selection

A descriptor selection was performed based on the importance of descriptors assessed by RF (computeAttributeImportance)^68^ implemented in the R program.^69^ The objective was to achieve an optimal QSAR model with the fewest possible descriptors. Optimisation of QSAR classification models was performed using ten-fold or Out-of-Bag (OOB) cross-validation methodology with the training set, employing subsequent statistical metrics including true positives (TP), true negatives (TN), false positives (FP), false negatives (FN), sensitivity (SE, prediction accuracy for active PD-1/PD-L1 inhibitors), specificity (SP, prediction accuracy for inactive PD-1/PD-L1 inhibitors), overall predictive accuracy (*Q*) and matthews correlation coefficient (MCC).

### 4.4. Balance of classes

The distribution of classes plays a very important role in classification models. The performance of machine learning algorithms can be significantly biased when the minority class, typically the one of interest, is underrepresented in the dataset.^70^ This scenario is evident in our PD-L1 activity training set, where there exists an imbalance ratio of 3 : 200 for the active/inactive classes, respectively. To address this issue, in the Random Forest method, the sampsize parameter in R program version 3.4.4 (ref. 69) was set to match the size as the less representative class, namely the active class. By adjusting this parameter, certain molecules from the minority class were utilized multiple times. For the SVM algorithm, the class weight parameter was adjusted to “balanced” to mitigate the impact of class imbalance.

### 4.5. ML techniques

#### 4.5.1. Random forests (RF)

A Random Forest (RF)^68,71^ is constructed as a collection of unpruned classification trees generated from bootstrap samples of the training set. In the construction of each individual tree, the optimal split at each node is determined using a randomly selected subset of descriptors. To ensure diversity in the ensemble, unique training and validation sets are used for the creation of each tree. Predictions are derived through a majority voting mechanism among the classification trees within the forest. To evaluate performance, the method employs internal assessment by calculating prediction errors for objects omitted in the bootstrap procedure, a process akin to internal cross-validation or OOB estimation. The approach quantifies descriptor importance based on the average decrease in impurity and the count of nodes utilizing a particular attribute.

Furthermore, RFs provide a probability assignment for each prediction, reflecting the level of confidence determined by the number of votes garnered by the predicted class. The RFs were built using the R program^69^ version 3.4.4, using the random forest library.^72^

#### 4.5.2. Support vector machines (SVM)

SVM^73^ employs nonlinear mapping to project the data into a hyperspace, where it establishes a boundary or hyperplane that effectively segregates the two categories of molecules: active and inactive. The positioning of this boundary relies on instances from the training set, commonly referred to as support vectors. When dealing with nonlinear data, kernel functions can be applied to transform it into a hyperspace, thereby rendering the classes linearly separable. In this study, SVMs were implemented using Scikit-learn^74^ and the LIBSVM package.^75^ The SVM type was configured as C-SVM-classification, employing the radial basis function as the kernel function. Hyperparameter tuning was conducted through ten-fold cross-validation with the GridSearchCV. The parameters *C* and *γ* of the CSVM-classification were optimized in the range of 1–50 and 0.0001–0.01 respectively 3.593813663804626 and 0.007742636826811269, while other parameters retained their default values. To address the issue of class imbalance, the class weight parameter was adjusted to “balanced,” ensuring replication of the smaller class until it matched the number of molecules in the larger class.

#### 4.5.3. Deep learning multilayer perceptron networks (_d_MLP)

Feed-forward neural networks were implemented through the open-source software library Keras^76^ version 2.2.5, utilizing the Tensorflow numerical backend engine.^77^ These extensively used ML algorithm, written in Python, simplify the development and application of deep neural networks. However, designing an appropriate network architecture poses a key challenge in employing _d_MLP. After conducting several experiments, the optimal hyperparameter settings for our study were selected through 10-fold cross-validation experiments with the training set, as outlined in Table 10.

**Table 10:** Hyperparameter settings of the best _d_MLP model

Hyperparameter	Setting
Initializer	Glorot uniform
Number of hidden layers	4
Number of neurons in the 1st, 2nd, 3rd and 4th layers	50
Activation 1st to 3rd layers	Relu
Activation 4th layer	Relu
Batch size	36
Optimizer	Adam
Loss	Binary crossentropy
Epochs	500

### 4.6. Molecular docking

Each of the 29 197 small organic molecules were docked to PD-L1, and the correlation between activity and binding energy against PD-L1 for each molecule was analysed. The software program OpenBabel (version 2.3.1) was used to convert the SDF files to PDBQT files. PDBQT files were used for docking to PD-L1 receptor (PDB ID: 5N2F, https://www.rcsb.org/structure/5N2F) with AutoDock Vina (version 1.1). Prior to docking, water molecules and ligands were removed from 5N2F using the AutoDockTools (http://mgltools.scripps.edu/). The search space coordinates were centered at *X*: 32.759, *Y*: 12.47, *Z*: 134.541; with dimensions of *X*: 20 000, *Y*: 20 000, *Z*: 20 000. Ligand tethering of the PD-L1 receptor was performed by regulating the genetic algorithm (GA) parameters, using 10 runs of the GA criteria. The resulting docking binding poses were visualised with PyMOL Molecular Graphics System, Version 2.0 Schrödinger, LLC, UCSF Chimera,^78^ and Protein–Ligand Interaction Profiler (PLIP) web tool.^79^ As a positive control test, both the inhibitor (*i.e.* BMS-200, Fig. 1) from the X-ray structure of the PD-L1/inhibitor complex and the same inhibitor with the 3D optimisation approach (*i.e.* JChem CXCALC) were docked. Docking scores of 29 197 small organic molecules against the PD-L1 protein are presented on Table S8, ESI.†

### 4.7. Biological activity evaluation

In the context of cancer treatment, PD-L1/PD-1 inhibitors harness the immune response of T cells against cancer cells. New approaches using small molecules are being pursued to overcome the limitations of mAbs and improve clinical responses.^42^ The biological activity of the proposed small molecules obtained by our approach was assessed by the ability to counteract the binding of PD-1 to PD-L1.

#### 4.7.1. Reagents

Sonidegib (BD328430, purity, ≥99.93%) and Lapatinib (BD220070, purity, ≥ 98%) were purchased from BLD Pharmatech (Laborspirit, Portugal) and were dissolved in dimethyl sulfoxide (DMSO; 472301; Sigma-Aldrich). The positive competitor control PD-1–PD-L1 inhibitor 1 (BMS1, PDI-1, BP158531, purity ≥98%) was purchased from Biosynth Ltd (Laborspirit, Portugal) and dissolved in Phosphatase-Buffered Saline (PBS). Purified recombinant monomeric protein human PD-L1His, the dimeric protein PD-1Fc chimera, and durvalumab were produced in plants.^61^ The monoclonal antibodies used were: mouse anti-human anti-CD274 (clone MIH1) conjugated with phycoerythrin (PE) fluorescent dye (cat no. 557 924. BD pharmingen) and anti-human IgG Fc – Horseradish Peroxidase (Merck millipore, catalog no. AP113P, USA). Due to solubility issues, only sonidegib was experimentally evaluated.

#### 4.7.2. Competitive ELISA

Inhibition of binding of soluble PD-1 to immobilized PD-L1 by putative blocker drug candidates was monitored using competitive ELISA assay. Binding of PD-1 to PD-L1 was assessed in the presence or the absence of the putative blocker at different concentrations. Serial dilutions were prepared in PBS-0.05% (v/v) Tween using the following protocol: for sonidegib, the stock concentration (100 mM, dissolved in DMSO) was submitted to 6 serial 10-fold dilutions (500–0.0005 μM); for PDI-1, the stock concentration (2.1 mM) was pre -diluted 2 folds followed by 3 serial 10-fold dilutions (1050–1.05 μM). The percentage of solvent DMSO never exceeded 0.5% (v/v).

Briefly, we used Corning®96 wells EIA/RIA assay microplates (Merck, Corning catalog no. 3590). Coating was performed by incubation of recombinant purified human PD-L1His in the wells overnight at 4 °C. Then, recombinant human PD-1Fc chimera protein and drugs were mixed and pre-incubated for 30 min at room temperature before being added to the wells and incubated for 2 hours at room temperature. After that, the microplate was incubated with Anti-human IgG Fc – Horseradish Peroxidase for 1 h and then washed. Then, 3,3′,5,5′-Tetramethylbenzidine (TMB) (Life technologies, cat no. 002023) was added for 2–3 minutes at room temperature. After coating, blocking was performed with PBS-0.05% (v/v) Tween containing 3% Bovine Serum Albumin (BSA). Between incubations, the wells were washed 5 times with PBS-0.05% Tween. The absorbance was measured at 450 nm and at 630 nm with mobi (μ2 MicroDigital) spectrophotometer.

#### 4.7.3. IC_50_ calculation

The inhibitory concentration (IC_50_) (concentration that causes 50% inhibition) was calculated based on dose response curves obtained by ELISA. The value was determined by analysing the log of the concentration–response curves by nonlinear regression analysis using the GraphPad Prism 8.0.1 (GraphPad Software, Inc., San Diego, CA, USA).

#### 4.7.4. Cell culture

To verify the effects on the interaction between PD-1 and PD-L1 when these proteins are expressed on living cells, we resorted to the human breast cancer cell line MDA-MB-231 (kindly provided by Professor Philippe Delannoy from the University Lille, France). These cells were grown in Dulbecco's modified Eagle medium (DMEM; Sigma), supplemented with 10% (v/v) foetal bovine serum (FBS; Gibco), 2 mM l-glutamine (Gibco), and 10 U mL^−1^ penicillin with 100 μg mL^−1^ streptomycin (Pen-Strep; Sigma). Cell cultures were kept in a humidified incubator at 37 °C with an atmosphere containing 5% CO_2_. Furthermore, the cells were routinely tested for mycoplasma contamination using MycoAlertTM kit (Lonza).

#### 4.7.5. Flow cytometry

To evaluate the PD-L1 expression in the MDA-MB231 cell line, 3 × 10^5^ cells were stained using the anti-human CD274 conjugated with phycoerythrin (PE) at 2.5 μg mL^−1^ and incubated for 30 min at 4 °C in the dark.

To assess the effect on mAb binding, the putative blocker drug candidates were pre-incubated with mAb anti-CD274 (PE) (2.5 μg mL^−1^) prior to the addition to MDA-MB231 cell line. Durvalumab was used at 2 μg mL^−1^ PDI-1 at 21 μM, PD-1Fc at 3.4 μg mL^−1^. Sonidegib was dissolved in PBS with 0.5% (v/v) DMSO (500 μM). As controls, experiments where the cells were incubated with the mAb anti-CD274 alone, one with PBS and other with PBS containing 0.5% (v/v) DMSO were performed. After completing the staining protocol, all cells were fixed with flow fix 2% paraformaldehyde fixative kit (Polysciences, Inc.) and the data was acquired in the Attune flow cytometer (ThermoFisher Scientific, USA). The data obtained was analysed using FlowJoTM v10.8.1 Software (BD Life Sciences).

#### 4.7.6. Statistical analysis

Statistical analysis was performed using the GraphPad Prism 8.0.1 (GraphPad Software, Inc., San Diego, CA, USA) and, unless otherwise stated, one-way ANOVA was used.

## Abbreviations

ADMET: Absorption–distribution–metabolism–excretion–toxicity
BMS: Bristol Myers Squibb
CADD: Computer-aided drug design
DC: Dendritic cell
_d_MLP: Deep learning multilayer perceptron networks
ELISA: Enzyme-linked immunosorbent assay
EMA: European Medicines Agency
FDA: Food and Drug Administration
FN: False negatives
FP: False positives
FPs: Fingerprints
HTS: High throughput screening
IC_50_: Concentration that causes 50% growth inhibition
ICIs: Immune checkpoint inhibitors
Ig: Immunoglobulin
InChI: International chemical identifier
Log*P*: The octanol–water partition coefficient
MCC: Matthews correlation coefficient (MCC)
MD: Molecular dynamics
ML: Machine learning
MW: Molecular weight
mAbs: Monoclonal antibodies
OOB: Out of bag
PBVS: Pharmacophore-based virtual screening
PDB: Protein data bank
*Q*
: Overall predictive accuracy (the ratio of the sum of true positive and true negative to the sum of true positive, true negative, false positive and false negative)
QSAR: Quantitative structure–activity relationship
R-o-5: Lipinski rule-of-five
RF: Random forest
SAR: Structure–activity relationship
SE: Sensitivity (the ratio of true positive to the sum of true positive and false positive)
SP: Specificity (the ratio of true negative to the sum of true negative and false negative)
SVM: Support vector machine
TN: True negatives
TP: True positives

## Data availability

All data generated or analysed during this study are included in the article and ESI.†

## Author contributions

Conceptualization: F. P., P. A. V. and Z. S.; methodology: F. P., P. A. V. and Z. S.; investigation: P. S. S., T. C., S. I., A. C., Z. S. and F. P.; resources: F. P. and P. A. V. ; data curation: P. S. S. and F. P.; writing – original draft preparation: P. S. S., T. C., Z. S. and F. P.; writing – review and editing: P. S. S., T. C., A. C., Z. S., P. A. V. and F. P.; project administration: F. P. and P. A. V.; funding acquisition: F. P. and P. A. V. All authors have read and agreed to the published version of the manuscript.

## Conflicts of interest

The authors declare that they have no conflict of interest.

## Supplementary Material

RA-015-D4RA08245A-s001

RA-015-D4RA08245A-s002

RA-015-D4RA08245A-s003
