# Supplementary material for: Advancements in drug discovery: integrating CADD tools and drug repurposing for PD-1/PD-L1 axis inhibition
Source: RSC Adv. 2025 Jan 23;15(4):2298–316. doi: 10.1039/d4ra08245a (PMC11755407; doi:10.1039/d4ra08245a)
Supplement: RA-015-D4RA08245A-s002 [file RA-015-D4RA08245A-s002.pdf]

This journal is © The Royal Society of Chemistry 2025

Table S1. PD-1 and PD-L1 FDA approved ICIs[1].

Target	ICIs	Brand name/ Marketed by	First approval date	Indications
PD-1	Pembrolizumab	Keytruda/ Merck	2014	Melanoma<br>Breast cancer<br>NSCLC[1]
	Nivolumab	Opdivo/ Bristol-Meyers Squibb	2014	Melanoma<br>NSCLC[1]<br>Head and neck cancer<br>Renal-cell cancer
	Cemiplimab	Libtayo/ Regeneron, Sanofi	2018	Cutaneous squamous-cell cancer<br>NSCLC[1]
	Dostarlimab	Jemperli/ GlaxoSmithKline LLC	2021	Endometrial cancer and recurrent or advanced<br>solid tumors
	Toripalimab	Loqtorzi/ Coherus Biosciences Inc.	2023	Melanoma
PD-L1	Atezolizumab	Tecentriq/ Genentech Inc., Roche	2016	Urothelial cancer
	Durvalumab	Imfinzi/ AstraZeneca	2017	Urothelial carcinoma<br>NSCLC[1]
	Avelumab	Bavencio/ Merck, Pfizer	2017	Urothelial carcinoma<br>Merkel-cell carcinoma<br>Renal-cell cancer

[1]Non-small cell lung cancer.

Table S2. Small molecule ICIs (3-8) targeting PD-1/PD-L1 in clinical trials[2].

Compound	Chemical structure	Clinical state	NCT Code	Indications
CA-170 (3)	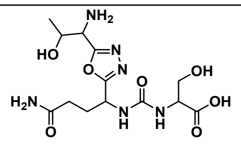	Phase 1	NCT02812875	Advanced solid tumors or lymphomas
INCB086550 (4)	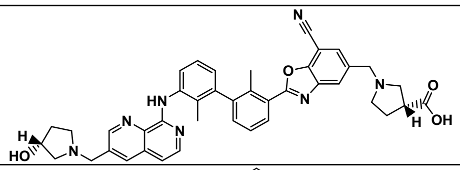	Phase 1<br>Phase 2	NCT03762447<br>NCT04629339	Solid tumors
IMMH-010 (5)	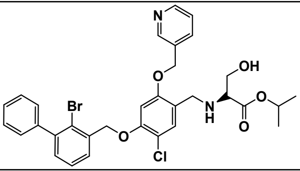	Phase 1	NCT04343859	Advanced solid tumors
MAX-10181 (6)	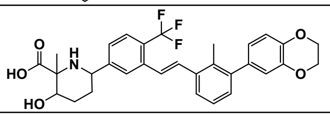	Phase 1	NCT05196360<br>NCT04122339	Advanced solid tumors

BPI-371153 (7)	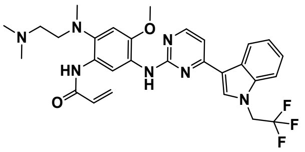	Phase 1	NCT05341557	Advanced Solid Tumors or Relapsed/ Refractory Lymphoma
ASC61 (8)	---[1]	Phase 1	NCT05287399	Advanced solid tumors

[1]Unknown structure.

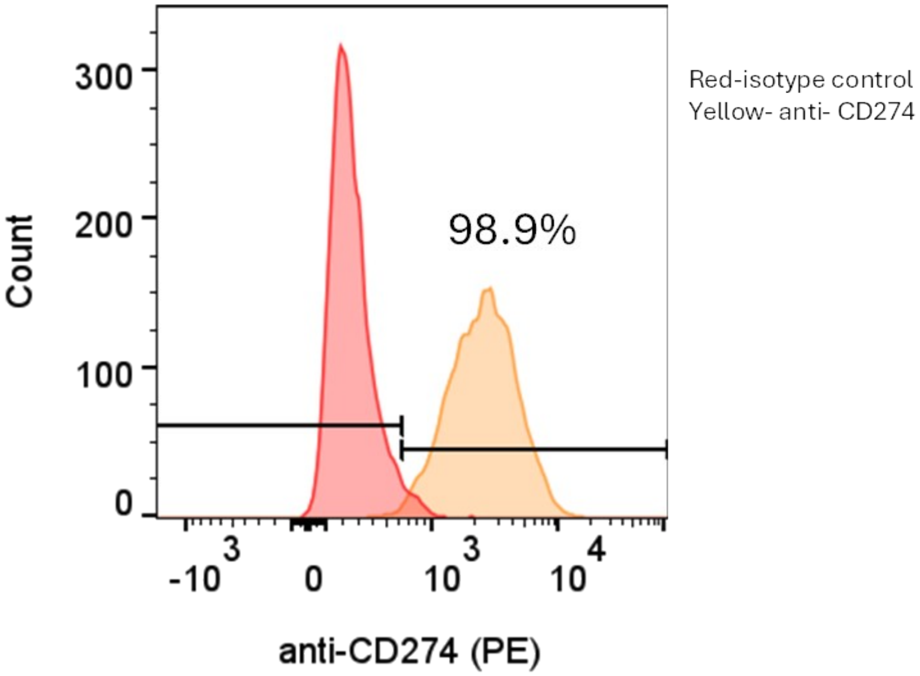

Fig. S1- MDA-MB-231 expresses PD-L1. Representative histogram from of MDA-MB-231 stained with anti-CD274 (PE) to assess PD-L1 expression levels by flow cytometry.

(1) *Drugs@FDA: FDA-Approved Drugs.* . 2024. https://www.fda.gov/drugs/development-approval-process-drugs/drug-approvals-and-databases (accessed May 2024).
(2) *The ClinicalTrials.gov results database*. https://clinicaltrials.gov/ (accessed 2024 May 2024).
